# Hookworm Infections and Sociodemographic Factors Associated With Female Reproductive Tract Infections in Rural Areas of the Central Region of Togo

**DOI:** 10.3389/fmicb.2021.738894

**Published:** 2021-11-03

**Authors:** Adjoa Holali Ameyapoh, Gnatoulma Katawa, Manuel Ritter, Christèle Nguepou Tchopba, Pélagie Edlom Tchadié, Kathrin Arndts, Hélène E. Kamassa, Bassimtou Mazou, Oukoe M. Amessoudji, Akawulu N’djao, Sibabe Agoro, Celina Vogelbusch, Millicent A. Omondi, Malewe Kolou, Simplice D. Karou, William Horsnell, Achim Hoerauf, Yaovi Ameyapoh, Laura E. Layland

**Affiliations:** ^1^Laboratoire de Microbiologie et de Contrôle de Qualité des Denrées Alimentaires, Unité de Recherche en Immunologie et Immunomodulation (UR2IM), Ecole Supérieure des Techniques Biologiques et Alimentaires (ESTBA), Université de Lomé, Lomé, Togo; ^2^Institute for Medical Microbiology, Immunology and Parasitology (IMMIP), University Hospital Bonn (UKB), Bonn, Germany; ^3^Hôpital du District de Tchaoudjo, Direction Préfectorale de la Santé, Sokodé, Togo; ^4^Direction Régionale de la Santé, Région la Kara, Kara, Togo; ^5^Division of Immunology, Faculty of Health Science, Institute of Infectious Disease and Molecular Medicine, University of Cape Town, Cape Town, South Africa; ^6^Faculté des Sciences de la Santé, Université de Lomé, Lomé, Togo; ^7^Wellcome Centre for Infectious Diseases Research in Africa (CIDRI), University of Cape Town, Cape Town, South Africa; ^8^Institute of Microbiology and Infection, University of Birmingham, Birmingham, United Kingdom; ^9^German Center for Infection Research (DZIF), Partner Site Bonn-Cologne, Bonn, Germany; ^10^German-West African Centre for Global Health and Pandemic Prevention (G-WAC), Partner Site, Bonn, Bonn, Germany

**Keywords:** female reproductive tract infections, risk factors, helminth infections, rural areas, central region of Togo

## Abstract

Female reproductive tract infections (FRTIs) have a huge impact on women’s health including their reproductive health in rural areas. Immunomodulation by helminth infections could influence the occurrence of FRTIs. This study aimed to investigate the association between FRTIs, hookworm infections, and sociodemographic factors in six rural areas of the central region of Togo. A semi-structured questionnaire was used to collect sociodemographical information, and parasitological assessments were used to diagnose helminth infections. Moreover, cytobacteriological examination of vaginal swabs was performed for the diagnosis of candidiasis and bacterial vaginosis (BV), and real-time PCR method was used to determine sexually transmitted infections (STIs). Finally, a logistic regression analysis was performed to assess the relationship and association of these factors to FRTIs. The prevalence of FRTIs was 82.3% including STIs (74.38%), BV (31.79%), and vulvovaginal candidiasis (9.85%). In detail, FRTIs were caused by bacteria such as *Ureaplasma parvum* (50%), *Ureaplasma urealyticum* (26.5%), and *Mycoplasma hominis* (17.5%) and viruses such us cytomegalovirus (5%) and human papilloma virus (HPV) (20%). No cases of *Haemophilus ducreyi*, *Treponema pallidum*, or varicella-zoster virus (VZV) were observed. Interestingly, women who had hookworm infections were at high risk of HPV. The use of condoms was a protective factor [adjusted odds ratio (aOR) = 0.23; 95% CI [0.11–0.51)], while the use of contraceptive methods was a risk factor [aOR = 2.49; 95% CI (1.19–5.19)] for STIs. The risk of BV was lower among participants who had more than four pregnancies [aOR = 0.27; 95% CI (0.11–0.65)]. Furthermore, women who had ever been paid for sexual intercourse were at high probability risk of vulvovaginal candidiasis [aOR = 16.92; 95% CI (1.46–196.48)]. This study highlighted risk factors associated with FRTIs, the control of which would help to reduce the incidence of these diseases. Health-care professionals could develop education and sensitization strategies based on these risk factors, and anti-hookworm treatment concepts may be taken into consideration to minimize the risk of HPV infections.

## Introduction

Female reproductive tract infections (FRTIs) have a high incidence among various gynecological diseases (Yan et al., 2018). FRTIs comprise (i) endogenous infection such as bacterial vaginosis (BV) and vulvovaginal candidiasis; (ii) sexually transmitted infections (STIs) such as syphilis, gonorrhea, and chlamydia; and (iii) iatrogenic infections (Moragianni et al., 2019). Worldwide, FRTIs are a major public health problem and affect predominantly young women (Torondel et al., 2018). It has been observed that the prevalence of these infections varies a lot between countries and even among regions belonging to the same country (Rabiu et al., 2010). Sequelae of untreated FRTIs affect the well-being of women, such as infertility, cervical cancer, ectopic pregnancy, stillbirth, and spontaneous abortion (Diadhiou et al., 2019). Interestingly, FRTIs seriously impact women in the Sub-Saharan African region causing around 85% infertility (Hussen et al., 2018). Helminth infections could expose the person to various infections, protect from auto-immune diseases, or decrease disease severity and influence vaccine efficacity (Moreau and Chauvin, 2010; Hartmann et al., 2019). Helminthic infections which are predominantly prevalent in Africa could therefore contribute to the establishment of infectious diseases (Arndts et al., 2015; Katawa et al., 2015; Ritter et al., 2018, 2019). The impact of helminthiasis on female reproductive health and associated diseases is widely documented. For instance, (i) *Wuchereria bancrofti* and *Schistosoma haematobium* infections were associated with an increased risk of HIV infection, and (ii) an increased prevalence of human papilloma virus (HPV) infection has been observed in the soil-transmitted helminths-endemic area of Peru (Chetty et al., 2020).

Both FRTIs and helminthic infections are co-endemic and have been shown to be highest in low- and middle-income countries (Chetty et al., 2020). Thus, this study was designed to investigate the association between FRTIs, hookworm infections, and sociodemographic factors in the central region of Togo.

## Materials and Methods

### Study Population and Ethics

This was a cross sectional study conducted in October 2019 in six villages of the central region of Togo, namely, Sakalaoudè, Tseve, Fazao, Sagbadai, Alheridè, and Kikimini. The sample size (367) was calculated using Schwartz formula *n* = Z^2^ P (1 - P)/d^2^ where Z, the accepted risk error, is 1.96; d, the precision, is 0.05; P, the prevalence. In the central region of Togo, the prevalence of STIs is estimated at 25% according to the socio-demographic survey of 2013–2014 (Enquête Démographique et de Santé du Togo 2013–2014, available online) (World Bank, 2017). So using these factors and the prevalence of 25%, the minimum sample size is 288. Therefore, 367 women, sexually active and premenopausal, aged between 18 and 56 years old, were enrolled. Pregnant and/or women positive for HIV were not included in this study. All participants gave their written consent to participate. This study was ethically approved by the “Comité de Bioéthique pour la Recherche en Santé (CBRS)” of the Togo Ministry of Health (N°26/2017/CBRS) and the Ethical Review Board of the University Hospital Bonn (Lfd. Nr. 273/16).

### Variables

Sociodemographic and helminth infections were assessed as risk factors.

For sociodemographic risk factors, a pre-tested and semi-structured questionnaire was used to collect sociodemographical data. The questionnaire was validated during a pilot study (Katawa et al., 2021) and administered by a trained medical laboratory technician in French or in a local language. Then, the participants who agreed moved freely to the sampling sites with a ticket proving their participation in the study. The probable associated risk factors chosen for analysis included helminth status, age, marital situation, duration of relationship with current partner, level of education, monthly income, average length of menstruation, vaginal hygiene methods, use of objects for intimate cleansing, introduction of products in vagina, having sex during menstruation, number of pregnancies, outcome of the last pregnancy, use of contraception methods, alcohol consumption, tobacco consumption, having sexual relationships during last 6 months, use of condoms, number of sexual partners during lifetime, use of condoms during last sexual intercourse, partner’s HIV positivity, having multiple sexual partners, having a sexual partner who has other sexual partners, and having paid intercourses/sexual relationships. Parasitological assessment was performed for the diagnosis of helminth infections. The confidentiality was maintained by an anonymization number that was attributed to each participant.

### Parasitological Assessment

For the diagnosis of helminth infections, stool, urine, and skin snips were collected. Using Kato Katz and urine sedimentation methods, helminths and *S. haematobium* eggs were determined in the stool and urine, respectively. In addition, two skin snips of 1–2 mm were taken from the left and right iliac crests using biopsy forceps. They were then incubated in 100 μl of NaCl (0.9%) in 96-well plates for 18–24 h at room temperature to detect the presence of *Onchocerca volvulus* microfilariae using microscopy.

### Reproductive Tract Infections Diagnosis

FRTIs were defined base on the Nugent score (>6) and the vaginal pH (>5).

### Candidiasis and Bacterial Vaginosis Diagnosis

Vaginal swabs were collected by physicians for cytobacteriological examination using fresh vaginal swab smears for yeast screening and gram staining for evaluation of bacterial flora by the criteria of Nugent (BV screening).

### Diagnosis of Female Reproductive Tract Infections

One vaginal swab and one cervical swab were collected by physicians from each participant and stored in 1 ml of eNAT^®^ medium (COPAN Italia S.P.A., Brescia, Italy). DNA was extracted using the Seegene Microlab Nimbus IVD automation (Seegene Inc., Seoul, South Korea) according to manufacturer’s instructions. Real-time PCR for 27 germs implicated in FRTIs and STIs was performed. Amplification was done on Bio-Rad CFX96 Real-time PCR System (Biorad) using the Allplex^TM^ STI Essential Assay kit that allows the simultaneous detection and identification of seven pathogens: *Chlamydia trachomatis*, *Mycoplasma genitalium*, *Mycoplasma hominis*, *Neisseria gonorrhoeae*, *Trichomonas vaginalis*, *Ureaplasma parvum*, and *Ureaplasma urealyticum*; the Allplex Genital Ulcer Assay kit that allows the detection of six pathogens: Cytomegalovirus (CMV), *Haemophilus ducreyi*, herpes simplex virus type 1 (HSV1), herpes simplex virus type 2 (HSV2), *Treponema pallidum*, and varicella-zoster virus (VZV); and the Anyplex II HPV HR detection kit that allows the detection of HPV infection. All reagents used for DNA extraction and FRTIs diagnosis were manufactured by Seegene Inc. (Seoul, Republic of Korea).

### Statistical Analysis

SPSS software (IBM SPSS Statistics 21; Armonk, NY) and GraphPad PRISM software 5.2 for Windows (GraphPap software, Inc., La Jolla, United States) were used to perform statistical analysis. Chi-square test was used to assess the prevalence differences (*p*-value threshold 0.05). Univariate and multivariate logistic regression analyses were used in order to investigate the risk factors associated with STIs, HPV, BV, and candidiasis including the calculation of odds ratios (OR) with 80% confidence intervals (CI) and adjusted odds ratios (aOR) with 95% CI. A threshold of *p*-value < 0.2 and < 0.05 were considered for univariate and multivariate analyses, respectively, and we declared the significance of OR/aOR if the value “1” is not included in the CI.

## Results

### Prevalence of Female Reproductive Tract Infections

**FIGURE 1:**
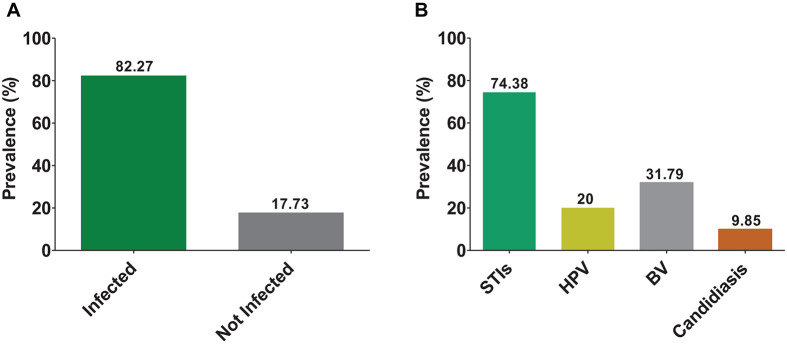
Prevalence of FRTIs. The bars indicate the prevalence of infected and non-infected **(A)**; and sexually transmitted infections (STI without HPV), human papilloma virus (HPV), bacterial vaginosis (BV), and candidiasis **(B)**.

The overall prevalence of FRTIs among women of the Central region of Togo was 82.3% (Figure 1). Considering the prevalence per village, we found a high prevalence at Sakalahoudè (64.29%) and Tcheve (50%).

**FIGURE 2:**
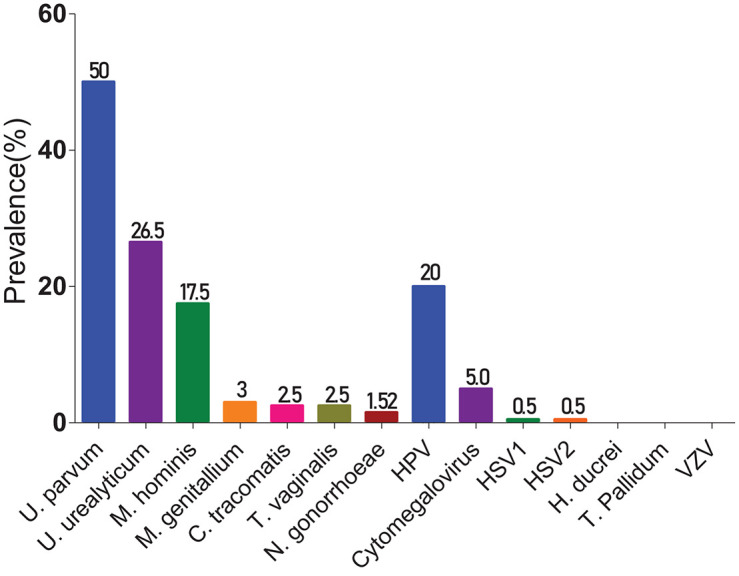
Prevalence of germs responsible for STIs. The bars indicate the prevalence of each germ involved in STIs.

Among all FRTIs screened, STIs were more prevalent (74.38%) than BV (31.79%) and candidiasis (9.85%) (Figure 1). The most infections implicated in STIs were caused by *U. parvum* (50% of women infected), followed by *U. urealyticum* (26.5%) and *M. hominis* (17.5%) (Figure 2). Viral infections detected included: HPV (20%) and CMV (5%). There were no cases of *H. ducreyi*, *T. pallidum*, or VZV. The prevalence of FRTIs in our study area was elevated with the predominance of hookworm infections. Therefore, we investigated the association between FRTIs and hookworm infections.

### Hookworm Infection Is Associated With Human Papilloma Virus Infection

**FIGURE 3:**
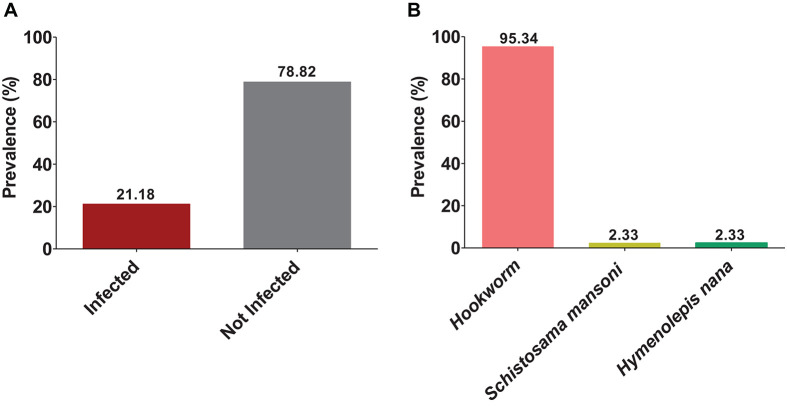
Prevalence of helminthiasis. The bars indicate the prevalence of infected and non-infected **(A)**. Prevalence of each species found on the cohort: hookworm, *Schistosoma mansoni*, and *Hymenolepis nana* **(B)**.

**FIGURE 4:**
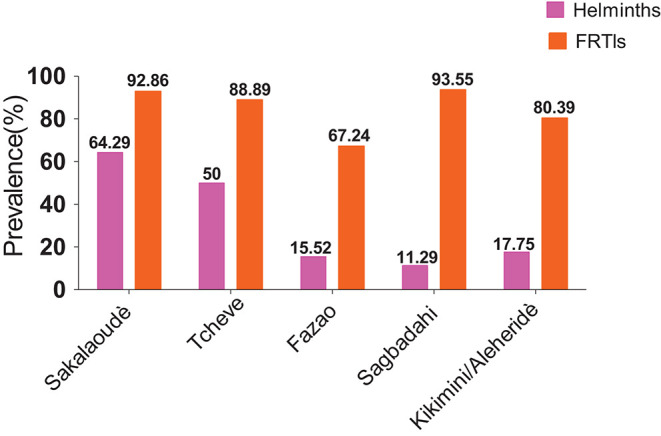
Prevalence of helminthiasis and FRTIs by village. The pink bars present the prevalence of helminthiasis, and the orange bars present the prevalence of FRTIs.

The overall prevalence of helminth infection in the central region of Togo was 21.18% (Figure 3). Helminthiases were most prevalent in Sagbadahi (93.55%), Sakalahoudè (92.86%), Tcheve (88.89%), and Kikimini/Aleheridè (80.39%) (Figure 4).

Hookworms were the most frequent helminthic infection (95.34%); *Schistosoma mansoni* and *Hymenolepis nana* were found, each on 2.33% of parasites that infected women.

Therefore, univariable logistic regression analysis was performed. We could not observe any association between hookworm infection and STIs [OR = 1.29; 80% CI (0.75–2.19)], HPV [OR = 2.22; 80% CI (1.32–3.75)], BV [OR = 0.86; 80% CI (0.53–1.41)], and candidiasis [OR = 0.98; 80% CI (0.47–2.09)]. Analysis revealed that hookworm infection was a risk factor of HPV infection [OR = 2.22; 80% CI (1.32–3.75)] (Table 1).

**TABLE 1:** Descriptive and logistic regression analysis of hookworm infection associated with FRTIs.

	Descriptive analysis			Logistic regression analysis	
	Chi-square			Univariate	
	Negative	Positive	*p*-value	OR (80% CI)	*p*-value
	n (%)	n (%)			
**STIs**					
Hookworm					
No	43 (82.7)	119 (78.8)	0.55	1	
Yes	9 (17.3)	32 (21.2)		1.29 (0.75–2.19)	0.55
**HPV**					
Hookworm					
No	130 (83.3)	27 (69.2)	**0.047**	1	
Yes	26 (16.7)	12 (30.8)		2.22 (1.32–3.75)	**0.05**
**BV**					
Hookworm					
No	104 (78.2)	50 (80.6)	0.69	1	
Yes	29 (21.8)	12 (19.4)		0.86 (0.53–1.41)	0.69
**Candidiasis**					
Hookworm					
No	146 (79.8)	16 (80)	1.00	1	
Yes	37 (20.2)	4 (20)		0.98 (0.47–2.09)	0.98

*Bold values are significant p-values.*

### Sociodemographic Factors Associated With Female Reproductive Tract Infections

Here we aimed to investigate the association between sociodemographic factors with FRTIs. Univariate logistic regression analysis revealed the independent risk factors associated with STIs (Table 2). The independent risk factors associated with low risk of STIs were the age ranged 33–39 years [OR = 0.51; 80% CI (0.29–0.89)], the fact of being faithful to the same partner for more than 10 years [OR = 0.57; 80% CI (0.36–0.88)], the use of water and soap for vaginal hygiene [OR = 0.48; 80% CI (0.31–0.75)], the use of objects (cotton or tissue) for vaginal cleansing [OR = 0.44; 95% CI (0.28–0.69)], the fact of having had more than four pregnancies [OR = 0.63; 80% CI (0.42–0.96)], and the use of condoms [OR = 0.41; 95% CI (0.27–0.62)].

**TABLE 2:** Descriptive and logistic regression analysis of factors associated with STIs.

	Descriptive analysis			Logistic regression analysis			
	Chi-square			Univariate		Multivariate	
	Negative	Positive	*p*-value	OR (80% CI)	*p*-value	aOR (95% CI)	*p*-value
	n (%)	n (%)					
**Age range (years)**			0.25				
[18–25]	12 (23.1)	42 (27.8)		1		1	
[26–32]	10 (19.2)	41 (27.2)		1.17 (0.63–2.17)	0.74	1.36 (0.47–3.92)	0.57
[33–39]	18 (34.6)	32 (21.2)		0.51 (0.29–0.89)	**0.12**	0.62 (0.17–2.25)	0.45
≥40	12 (23.1)	36 (23.8)		0.86 (0.47–1.56)	0.74	1.22 (0.30–4.88)	0.78
**Duration of relationship with current partner (years)**							
≤10	21 (40.4)	80 (53.0)	0.25	1		1	
>10	25 (48.1)	54 (35.8)		0.57 (0.36–0.88)	**0.09**	0.40 (0.08–2.15)	0.29
Do not know	6 (11.5)	17 (11.3)		0.74 (0.37–1.48)	0.58	0.44 (0.09–2.14)	0.31
**Vaginal hygiene methods**			0.16				
Water	22 (42.3)	91 (60.3)		1		1	
Water and antiseptics	3 (5.8)	6 (4.0)		0.48 (0.19–1.26)	0.33	0.71 (0.19–2.63)	0.61
Water and detergent	3 (5.8)	6 (4.0)		0.48 (0.19–1.26)	0.33	0.67 (0.23–1.96)	0.46
Water and soap	24 (46.2)	48 (31.7)		0.48 (0.31–0.75)	**0.04**	0.71 (0.19–2.63)	0.61
**Objects used for intimate cleansing**							
No	14 (26.9)	69 (45.7)		1		1	
Yes	38 (73.1)	82 (54.3)		0.44 (0.28–0.69)	**0.02**	0.48 (0.19–1.15)	0.09
**Number of pregnancies**			0.157				
≤4	28 (53.8)	98 (64.9)		1		1	
>4	24 (46.2)	53 (35.1)		0.63 (0.42–0.96)	**0.16**	0.53 (0.19–1.50)	0.23
**Contraception**			**0.029**				
No	29 (55.8)	58 (38.4)		1		1	
Yes	23 (44.2)	93 (61.6)		2.02 (1.33–3.07)	**0.03**	2.49 (1.19–5.19)	**0.02**
**Alcohol consumption**			0.12				
No	49 (94.2)	130 (86.1)		1		1	
Yes	3 (5.8)	21 (13.9)		2.64 (1.16–5.99)	**0.13**	(0.59–9.53)	0.23
Use of condoms			**0.006**				
No	22 (53.8)	112 (74.2)		1		1	
Yes	24 (46.2)	39 (25.8)		0.41 (0.27–0.62)	**0.007**	0.23 (0.11–0.51)	**0.000**
**Number of partners during lifetime**			0.17				
≤1	41 (78.8)	104 (68.9)		1		1	
>1	11 (21.2)	47 (31.1)		1.68 (1.03–2.75)	**0.17**	1.46 (0.61–3.46)	0.39

*Bold values are significant p-values.*

The independent risk factors associated with high risk of STIs were the use of contraceptive methods [OR = 2.02; 80% CI (1.33–3.07)], alcohol consumption [OR = 2.64; 80% CI (1.16–5.99)], and the fact of having had more than one sexual partner in their lifetime [OR = 1.68; 80% CI (1.03–2.75)].

After controlling for confounding parameters through multivariable logistic regression analysis, adjusting independent factors linked to STIs, we found that the use of condoms was a protective factor [aOR = 0.23; 95% CI (0.11–0.51)], while the use of contraceptive methods was a risk factor [aOR = 2.49; 95% CI (1.19–5.19)].

As shown in Table 3, independent factors associated with low risk of HPV infections were the age ranged 26–32 years [OR = 0.47; 80% CI (0.23–0.94)], a live birth at last pregnancy [OR = 0.15; 80% CI (0.05–0.49)], the use of condoms [OR = 0.15; 80% CI (0.07–0.33)], and the fact of knowing that the current partner has other sexual partners [OR = 0.54; 80% CI (0.32–0.90)]. The risk of HPV infection was the highest in unmarried women or women not in a relationship [OR = 5.83; 80% CI (2.13–15.97)], who had more than one partner during their lifetime [OR = 2.66; 80% CI (1.65–4.28)], who had multiple partners [OR = 7.50; 80% CI (2.85–18.72)], and who did not know if their partner had other sexual partners [OR = 2.45; 80% CI (1.09–5.51)].

**TABLE 3:** Descriptive and logistic regression analysis of factors associated with HPV.

	Descriptive analysis			Logistic regression analysis			
	Chi-square test			Univariate		Multivariate	
	Negative	Positive	*p*-value	OR (80% CI)	*p*-value	a OR (95% CI)	*p*-value
	n (%)	n (%)					
**Age range**			0.1				
[18–25]	40 (25.6)	12 (30.8)		1		1	
[26–32]	43 (27.6)	6 (15.4)		0.47 (0.23–0.94)	**0.16**	0.52 (0.16–1.75)	0.29
[33–39]	41 (26.3)	7 (17.9)		0.57 (0.29–1.12)	0.28	0.52 (0.16–1.70)	0.28
≥40	32 (20.5)	14 (35.9)		1.46 (0.81–2.63)	0.41	1.12 (0.40–3.16)	0.82
**Marital situation**			**0.03**				
Married/in relationship	153 (98.1)	35 (89.7)		1		1	
Not married/not in relationship	3 (1.9)	4 (10.3)		5.83 (2.13–15.97)	**0.03**	2.59 (0.31–21.42)	0.38
**Vaginal hygiene methods**			0.12				
Water	88 (56.4)	19 (48.7)		1			
Water and antiseptics	8 (5.1)	0 (0)		0	0.99		
Water and detergent	5 (3.2)	4 (10.3)		3.71 (1.48–9.29)	0.99		
Water and soap	55 (35.3)	16 (41.0)		1.35 (0.83–2.19)	0.07		
**Outcome of the last pregnancy**			**0.02**				
Never get pregnant	2 (1.3)	3 (7.7)		1		1	
Live birth	152 (97.4)	34 (87.2)		0.15 (0.05–0.49)	**0.04**	0.10 (0.01–1.11)	0.06
Miscarriage	2 (1.3)	2 (5.1)		0.67 (0.12–3.78)	0.77	0.78 (0.03–20.84)	0.88
**Alcohol consumption**			0.26				
No	140 (89.7)	32 (82.1)		1		1	
Yes	16 (10.3)	7 (17.9)		1.92 (1.02–3.60)	**0.19**	0.96 (0.29–3.11)	0.94
**Use of condoms**			**0.001**				
No	100 (64.1)	36 (92.3)		1		1	
Yes	56 (35.9)	3 (7.7)		0.15 (0.07–0.33)	**0.002**	0.13 (0.03–0.49)	**0.003**
**Number of partners during lifetime**			**0.007**				
≤1	118 (75.6)	21 (53.8)		1		1	
>1	38 (24.4)	18 (46.2)		2.66 (1.65–4.28)	**0.008**	2.56 (1.10–5.96)	**0.03**
**Multiple partners**			**0.009**				
No	153 (98.1)	34 (87.2)		1		1	
Yes	3 (1.9)	5 (12.8)		7.5 (2.85–19,072)	**0.008**	7.14 (0.99–51.19)	0.05
**Partner who has other partners**			0.05				
No	79 (50.6)	23 (59.0)		1		1	
Do not know	7 (4.5)	5 (12.8)		2.45 (1.09–5.51)	**0.16**	0.83 (0.15–4.52)	0.83
Yes	70 (44.9)	11 (28.2)		0.54 (0.32–0.90)	**0.12**	0.44 (0.18–1.08)	0.07

*Bold values are significant p-values.*

Following the multivariate logistic regression analysis, the use of condoms [aOR = 0.13; 95% CI (0.03–0.49)] appeared to be a protective factor against HPV infections. while having more than one partner during the lifetime [aOR = 2.79; 95% CI (1.32–5.95)] appeared as a risk factor for HPV infections.

In Table 4, the univariate regression analysis revealed that the risk of BV infections was low among participants who had more than four pregnancies [OR = 0.49; 80% CI (0.32–0.75)], had a miscarriage in their last pregnancy [OR = 0.08; 80% CI (0.01–0.65)], had a live birth at their last pregnancy [OR = 0.11; 80% CI (0.26–0.47)], and used condoms [OR = 0.45; 80% CI (0.28–0.73)]. The risk probability of BV was high among women who were not married or not in a relationship [OR = 3.80; 80% CI (1.46–9.91)], did not know the length of their relationship with their current partner [OR = 3.00; 80% CI (1.63–5.53)], did not know if their sexual partner was HIV positive [OR = 1.82; 80% CI (1.22–2.70)], and had multiple partners [OR = 3.80; 80% CI (1.46–9.91)].

**TABLE 4:** Descriptive and logistic regression analysis of factors associated with BV.

	Descriptive analysis			Logistic regression analysis			
	Chi-square test			Univariate		Multivariate	
	Negative	Positive	*p*-value	OR (80% CI)	*p*-value	aOR (95% CI)	*p*-value
	n (%)	n (%)					
**Marital situation**			0.06				
Married/in relationship	130 (97.7)	57 (91.9)		1		1	
Not married/not in relationship	3 (2.3)	5 (8.1)		3.80 (1.46–9.91)	**0.07**	0.89 (0.11–6.99)	0.91
**Duration of relationship with current partner (years)**			**0.02**				
≤10	67 (50.4)	29 (46.8)		1		1	
>10	56 (42.1)	20 (32.2)		0.83 (0.53–1.28)	0.58	4.59 (1.26–16.72)	**0.02**
Do not know	10 (7.5)	13 (21.0)		3.00 (1.63–5.53)	**0.02**	1.67 (0.69–4.02)	0.25
**Number of pregnancies**			**0.04**				
≤4	76 (57.1)	45 (72.6)		1		1	
>4	57 (42.9)	17 (27.4)		0.49 (0.32–0.75)	**0.03**	0.27 (0.11–0.66)	**0.004**
**Outcome of the last pregnancy**			**0.07**				
Never get pregnant	1 (0.8)	4 (6.5)		1		1	
Live birth	129 (97.0)	57 (91.9)		0.11 (0.26–0.47)	**0.05**	0.21 (0.02–2.17)	0.19
Miscarriage	3 (2.2)	1 (1.6)		0.08 (0.01–0.65)	**0.12**	0.14 (0.004–4.50)	0.27
**Use of condoms**			**0.03**				
No	87 (65.4)	50 (80.6)		1		1	
Yes	40 (34.6)	12 (19.4)		0.45 (0.28–0.73)	**0.03**	0.46 (0.21–0.99)	0.05
**Partner HIV positivity**			0.08				
No	77 (57.9)	27 (43.5)		1		1	
Do not know	55 (41.4)	35 (56.5)		1.82 (1.22–2.70)	**0.07**	1.71 (0.87–3.36)	0.12
Yes	1 (0.7)	0 (0)		0	1.00	0	1.00
**Multiple partners**			0.11				
No	130 (97.7)	57 (91.9)		1		1	
Yes	3 (2.3)	5 (8.1)		3.80 (1.46–9.91)	**0.07**	2.17 (0.37–12.85)	0.39

*Bold values are significant p-values.*

After adjusting the variable associated with BV, the risk of BV infections was low among participants who had a number of pregnancies superior to 4 [aOR = 0.27; 95% CI (0.11–0.66)].

The high risk of candidiases was observed among women aged between 26 and 32 years old [OR = 4.84; 80% CI (1.69–13.78)], women older than 40 years [OR = 3.71; 80% CI (1.26–10.93)], who earned less than 70 US dollars [OR = 4.08; 80% CI (1.07–15.56)], used objects (tissue or cotton) for vaginal cleansing [OR = 2.23; 80% CI (1.12–4.44)], had multiple partners [OR = 3.28; 80% CI (1.09–9.78)], did not know if their partner had other partners [OR = 2.88; 80% CI (1.12–7.42)], and who had ever been paid for sex [OR = 4.97; 80% CI (1.57–15.77)]. Finally, using a backward stepwise logistic regression analysis, in a model with age range, monthly income, use of objects for intimate cleansing, practice of paid sexual intercourse, having current multiple partners, having a partner who has other partners, and practicing paid sexual intercourse, it appeared that women who practiced paid sexual intercourse were at high probability risk of candidiasis [aOR = 16.92; 95% CI (1.46–196.48)] (Table 5).

**TABLE 5:** Descriptive and logistic regression analysis of factors associated with candidiasis.

	Descriptive analysis			Logistic regression analysis			
	Chi-square			Univariate		Multivariate	
	Negative	Positive	*p*-value	OR (80% CI)	*p*-value	a OR (95% CI)	*p*-value
Candidiasis	n (%)	n (%)					
**Age range**			0.19				
[18–25]	52 (28.4)	2 (10.0)		1			
[26–32]	43 (23.5)	8 (40.0)		4.84 (1.69–13.78)	**0.05**		
[33–39]	46 (25.1)	4 (20.0)		2.26 (0.72–7.07)	0.36		
≥40	42 (23.0)	6 (30.0)		3.71 (1.26–10.93)	**0.12**		
**Monthly income**			0.36				
≤70 US Dollars	149 (81.4)	19 (95.0)		4.08 (1.07–15.56)	**0.17**	10.14 (0.76–135.32)	0.08
>70 US Dollars	2 (1.1)	0 (0)		0	1.00	0	1.00
Do not earn money	32 (17.5)	1 (5.0)		1		1	
**Objects used for intimate cleansing**			0.13				
No	78 (42.6)	5 (25)		1			
Yes	105 (57.4)	15 (75)		2.23 (1.12–4.44)	**0.14**		
**Multiple partners**			0.14				
No	177 (96.7)	18 (90.0)		1			
Yes	6 (3.3)	2 (10.0)		3.28 (1.09–9.78)	**0.16**		
**Partner who has other partners**			0.36				
No	95 (51.9)	9 (45.0)		1			
Do not know	11 (6.0)	3 (15.0)		2.88 (1.12–7.42)	**0.15**		
Yes	77 (42.1)	8 (40.0)		1.09 (0.57–2.11)	0.86		
**Paid sexual intercourse**			0.11				
No	179 (97.8)	18 (90.0)		1		1	
Yes	4 (2.2)	2 (10.0)		4.97 (1.57–15.77)	**0.08**	16.92 (1.46–196.48)	**0.02**

*Bold values are significant p-values.*

## Discussion

This study revealed that STIs were predominantly prevalent (74.38%) in six rural villages of the central region of Togo. Serious complications of STIs are ectopic pregnancy, pelvic inflammatory disease, preterm labor, miscarriage, stillbirth, and congenital infection, and they may lead to chronic disability (such as infertility and genital cancer) and death. Here, we diagnosed 27 germs incriminated in STIs. In detail, *U. parvum* was the most prevalent affecting 50% of women, followed by *U. urealyticum* (26.5%) and *M. hominis* (17.5%). Those three species belong to the gender *Mycoplasma*, a gender of bacteria frequently isolated from the genital tract of both men and women (Kokkayil and Dhawan, 2015). In Italy, Leli et al. (2018) found that *U. parvum* was the most isolated mycoplasma among women with a prevalence of 38.3%, and a study in South Africa revealed that *U. parvum* was isolated from 72.4% vaginal samples of pregnant women (Redelinghuys et al., 2013). In general, *U. parvum* is associated with intrauterine inflammation (Kasper et al., 2010).

Among viruses found in the cohort, human CMV was predominately prevalent (5%). In Africa, CMV is a neglected pathogen, but its burden is important with a seroprevalence close to 100% (Bates and Brantsaeter, 2016). CMV is a member of *Herpesviridae* family and is classified as an emerging STI (Kent, 2017) that is transmitted through direct contact with multiple fluids of the body like saliva, urine, milk, and genital secretions. In addition, it can also be transmitted from an infected mother to her fetus (CDC, 2020b). The virus can stay latent in infected persons until the immune system weakening allows the virus to reactivate (Grinde, 2013).

In regards to risk factors, this study revealed that the use of contraceptive methods appeared as a high risk of STIs. Contraceptives are used for birth control and have a socio-economic impact. According to Deese et al. (2018) some contraceptive methods have related to STIs, but robust studies should be conducted to provide accurate information and facilitate contraceptive choice by women. Moreover, the presence of STIs was also associated with alcohol consumption, confirming other studies showing that increased risk of STIs was influenced by alcohol drinking across a wide variety of populations (Arasteh et al., 2008; Hutton et al., 2008). That is justified by the fact that alcohol drinking can lead to immune deficiency and increase sensitivity to infectious diseases (Zitkute and Bumbuliene, 2016). Another independent factor associated with STIs was the type of relationship and sexual behavior, especially the number of sexual partners during the lifetime, confirming a study in Brazil among rural women, which revealed that a higher number of partners in life was a risk factor for STIs (Oliveira et al., 2008). In addition, having had more than one partner during the lifetime appeared as a high-risk factor for HPV infections, confirming a previous study from Molano et al. (2002) who found that having had more than one regular sexual partner increased HPV infection risk (OR 1.9).

In general, HPV plays an important role as a leading cause of most cervical cancers. More than 100 types of HPV have been identified and at least 14 of them are associated as risk factors for cancer and especially 2 types (HPV 16 and HPV 18) cause 70% of cervical cancers and pre-cancerous cervical lesions (WHO, 2019). Nevertheless, in our cohort, HPV 16 and HPV 18 were less prevalent (respectively, 1.54 and 1.03%), but associations to cancer diseases need to be investigated in future studies.

Interestingly, the impact of helminth immune regulation on susceptibility to STIs is known. Given the global incidence of helminths and their detrimental impact on public health (WHO, 2017), the geographical overlap between helminth exposure and STIs might be a result of parasite-induced changes on female reproductive health (Gopinath et al., 2000; Wolday et al., 2002) and helminth-induced immunomodulation (Brown et al., 2005). Indeed, here we showed that women who had hookworm infections had a 2.22 times risk of HPV infection than those who were not infected by hookworm. Gravitt et al. (2015) found that women infected with soil-transmitted helminths (STHs) had 60% higher prevalence of HPV, compared with those without STHs infection with a prevalence ratio of 1.6. The helminth-induced Th2 response in vagina could explain the plausibility of high HPV occurrence (Gravitt et al., 2015).

Concerning endogenous infections, 31.79% of women were affected by BV and 9.85% had candidiasis. In Sokodé, the principal city of the central region of Togo, Tchelougou et al. (2013) found on pregnant women a prevalence of 55.31% of *Gardnerella vaginalis* and 50.77% of candidiasis, which is caused by *Candida* species and has been shown to be responsible for inflammatory changes in the vaginal and vulvar epithelium (Jeanmonod and Jeanmonod, 2020). However, the differences between the studies could be due to pregnancy status with unbalance vaginal flora.

In addition, BV, which is the most common vaginal condition in women aged between 15 and 44 years (CDC, 2020a), is known to increase predisposition to STIs (Lata et al., 2010; Bautista et al., 2017). In detail, it is characterized by an altered vaginal microflora including an increase in the vaginal pH, reduced lactobacilli species, and increased hydrogen peroxide producing species and facultative and anaerobic bacteria numbers and/or types (Eschenbach et al., 2000).

Interestingly, the risk probability of BV was high among those who were not married or not in a relationship and those who had multiple partners. Ranjit et al. (2018) found that 100% of unmarried women were more prone to BV, and indeed, another study revealed that BV has been associated with having three or more male sexual partners in the past 12 months (OR = 1.60, 95% CI: 1.19–2.04) (Smart et al., 2004). Moreover, the number of pregnancies more than 4 was a protective factor against BV infections. This was in line with a study conducted in Ghana where Konadu et al. (2019) found that the fact of having more than four pregnancies was protective for BV.

In a logistic regression model with monthly income and practicing paid sexual intercourse, we found that women who practiced paid sexual intercourse were at high probability risk of candidiasis. Unlike our results, Konadu et al. (2019) did not find any possible risk factor associated with candidiasis, although they did not include the practice of paid sexual intercourse as a possible risk factor. We can notice that the study population of Konadu et al. (2019) was pregnant women attending antenatal clinic, whereas our study population was exclusively non-pregnant women.

Finally, the use of condoms was a protective factor against STIs, confirming a previous study (Ginindza et al., 2017). Moreover, several studies revealed that consistent condom use was also associated with significantly decreased risk of gonorrhea, chlamydia, genital ulcer disease, BV, HPV, and pelvic inflammatory disease but did not have an impact on candidiasis (Baeten et al., 2001).

The present study had some limitations including the final samples size and one stool sample per participant.

## Conclusion

This study depicted a high prevalence of FRTIs in six villages of the central region of Togo. Among them, STIs were predominately prevalent, and factors such as helminth infection, the use of contraceptives, the number of pregnancies, the number of partners, the monthly income, the methods of cleansing, the marital status, and alcohol drinking were associated with the occurrence of FRTIs. Local healthcare authorities must focus on women education and sensitization strategies based on these risk factors to optimize prevention and control measures against FRTIs.

## Data Availability Statement

The raw data supporting the conclusions of this article will be made available by the authors, without undue reservation.

## Ethics Statement

The studies involving human participants were reviewed and approved by the Comité de Bioéthique pour la Recherche en Santé (CBRS) du ministère de la santé du Togo. The patients/participants provided their written informed consent to participate in this study.

## Author Contributions

GK, AHA, CNT, PT, CV, AN’d, SA, HK, BM, and OA carried out the survey and the field work. CV, MR, GK, AHA, MO, CNT, and PT analyzed the samples. GK, MR, LEL, AH, WH, MK, YA, and SK designed the study protocol, analyzed, interpreted, and validated all the data. GK, WH, MR, CNT, and LEL wrote the manuscript which was then read and approved by all other authors. All authors contributed to the article and approved the submitted version.

## Conflict of Interest

The authors declare that the research was conducted in the absence of any commercial or financial relationships that could be construed as a potential conflict of interest.

## Publisher’s Note

All claims expressed in this article are solely those of the authors and do not necessarily represent those of their affiliated organizations, or those of the publisher, the editors and the reviewers. Any product that may be evaluated in this article, or claim that may be made by its manufacturer, is not guaranteed or endorsed by the publisher.
